# In situ split plus portal vein ligation (ISLT) – a salvage procedure following inefficient portal vein embolization to gain adequate future liver remnant volume prior to extended liver resection

**DOI:** 10.1186/s12893-020-00721-y

**Published:** 2020-04-06

**Authors:** Nadja Lehwald-Tywuschik, Sascha Vaghiri, Jan Schulte am Esch, Salman Alaghmand, Yan Klosterkemper, Lars Schimmöller, Anja Lachenmayer, Hany Ashmawy, Andreas Krieg, Stefan A. Topp, Alexander Rehders, Wolfram Trudo Knoefel

**Affiliations:** 1 Department of Surgery A, University Hospital Duesseldorf, Duesseldorf, Germany; 2 Department of General, Visceral, Thorax and Pediatric Surgery,Heinrich-Heine-University Hospital, Moorenstr. 5, 40225 Duesseldorf, Germany; 3 Present address: Center of Visceral Medicine, Department of Visceral Surgery, Protestant Hospital of Bethel Foundation, Bielefeld, Germany; 4 Department of Diagnostic and Interventional Radiology, University Hospital Duesseldorf, Duesseldorf, Germany; 5 Present ccaddress: Department of Visceral Surgery and Medicine, University Hospital Bern, University of Bern, Bern, Switzerland; 6 Present address: Department of Surgery, Ameos Hospital, Bremerhaven, Germany

**Keywords:** ALPPS, Liver resection, In situ split, Future liver remnant, Liver hypertrophy

## Abstract

**Background:**

Right extended liver resection is frequently required to achieve tumor-free margins. Portal venous embolization (PVE) of the prospective resected hepatic segments for conditioning segments II/III does not always induce adequate hypertrophy in segments II and III (future liver remnant volume (FLRV)) for extended right-resection. Here, we present the technique of in situ split dissection along segments II/III plus portal disruption to segments IV-VIII (ISLT) as a salvage procedure to overcome inadequate gain of FLRV after PVE.

**Methods:**

In eight patients, FLRV was further pre-conditioned following failed PVE prior to hepatectomy (ISLT-group). We compared FLRV changes in the ISLT group with patients receiving extended right hepatectomy following sufficient PVE (PVEres-group). Survival of the ISLT-group was compared to PVEres patients and PVE patients with insufficient FLRV gain or tumor progress who did not receive further surgery (PVEnores-group).

**Results:**

Patient characteristics and surgical outcome were comparable in both groups. The mean FLRV-to-body-weight ratio in the ISLT group was smaller than in the PVEres-group pre- and post-PVE. One intraoperative mortality due to a coronary infarction was observed for an ISLT patient. ISLT was successfully completed in the remaining seven ISLT patients. Liver function and 2-year survival of ~ 50% was comparable to patients with extended right hepatectomy after efficient PVE. Patients who received a PVE but who were not subsequently resected (PVEnores) demonstrated no survival beyond 4 months.

**Conclusion:**

Despite extended embolization of segments I and IV-VIII, ISLT should be considered if hypertrophy was not adequate. Liver function and overall survival after ISLT was comparable to patients with trisectionectomy after efficient PVE.

## Background

To date, surgical hepatic resection is the only curative treatment for patients with primary or secondary liver malignancies that can achieve complete tumor removal with tumor-free resection margins. However, the extend of liver resection remains a challenging factor that is limited by the size of the future liver remnant volume (FLRV). In the past, extended liver resection even with a sufficient FLRV has been feared due to the increased risk of postoperative liver failure [1–5]. In the past years, much progress has been made to induce hypertrophy in the FLRV before major hepatectomy [6]. Portal vein embolization (PVE) represents a well-established standard treatment to increase the FLRV by up to 40% [7–10]. However, it usually takes up to 6–8 weeks after PVE to achieve an adequate liver volume for extended liver surgery; this interval can be as long as 150 days [6, 10]. Moreover, it was reported that PVE has a failure rate of 20–30% as a result of tumor progression or inadequate hypertrophy [8, 11–14]. In cases where PVE does not induce timely hypertrophy, the risk of tumor progression increases while awaiting sufficient FLRV to assure a safe extended liver resection [12, 13, 15].

Recently, a new technique for hepatic resection called in situ split dissection along segments II and III plus portal disruption to hepatic segments IV to VIII (ISLT) also called ALPPS (Associating Liver Partition and Portal Vein Ligation for Staged Hepatectomy) has been introduced to induce an accelerated response of the FLRV within a reasonable period of time [16–22]. To date, only a few reports with small patient numbers exist that describe various ISLT procedures as a rescue strategy to overcome failed “classical” portal occlusion measures; these include interventional embolization and open surgical portal ligation concepts. The various ISLT techniques were developed to provide an alternative approach to induce adequate FLRV growth while preserving the perspective of a potential curative surgical strategy [4, 5, 21, 23–26]. Here, we present a large single-center experience with ISLT as a salvage procedure for inefficient PVE of segments I/IV-VIII as a last measure to gain adequate FLRV.

### Methods

#### Patients

**Fig. 1:**
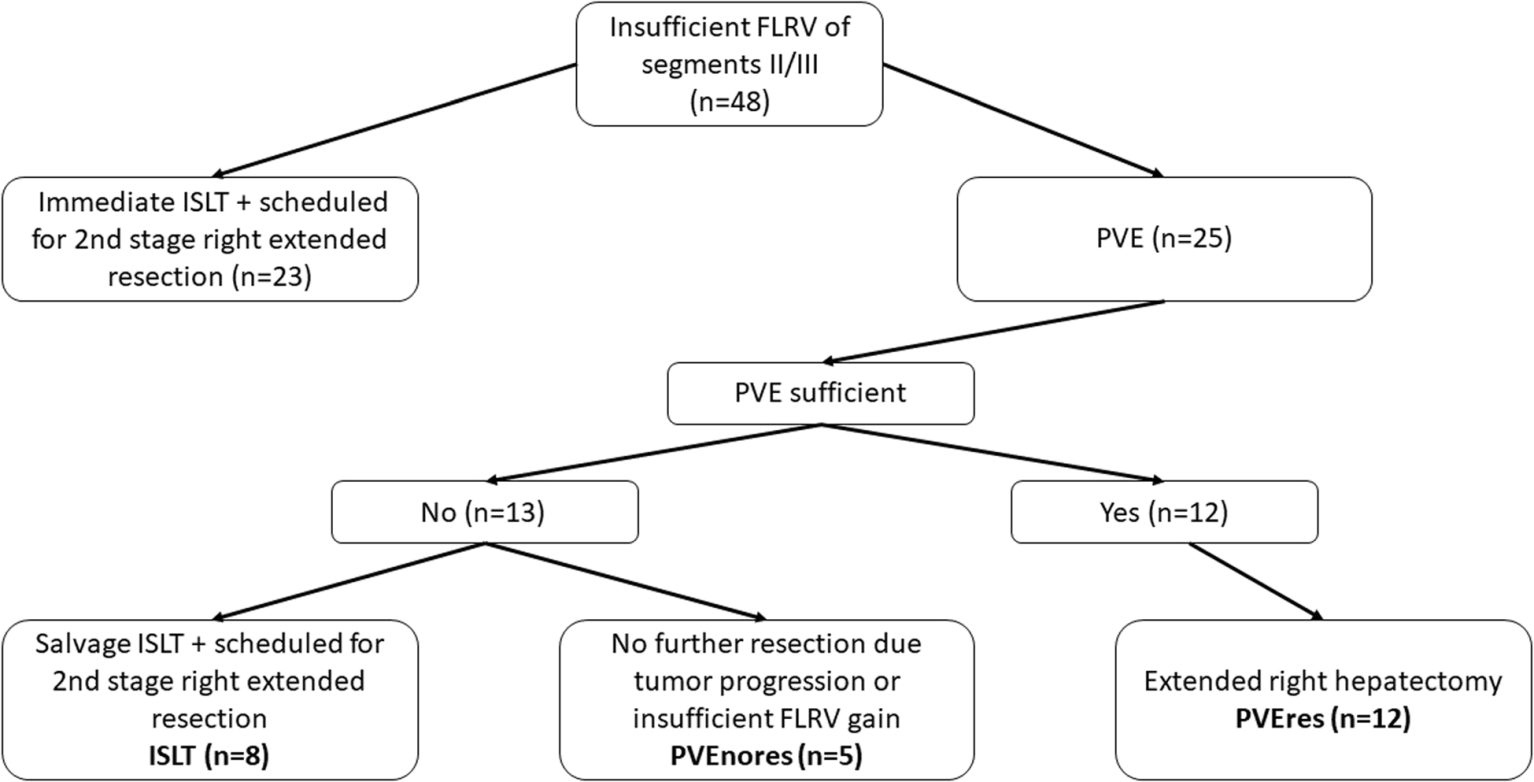
Flow chart of patient collective

Between January 2009 and January 2019, medical records of 48 consecutive patients scheduled for extended right hepatectomy (resection of hepatic segments I + IV to VIII) for primary or secondary liver malignancies were reviewed from the prospective clinical tumor registry, which is maintained by our oncological liver surgery program at the Department of General, Visceral, Thorax and Pediatric Surgery, University Hospital Duesseldorf, Germany. All of these patients were initially judged to be unresectable due to an insufficient FLRV (cumulative volume of segments II and III). Out of this cohort, 23 patients were scheduled for immediate ISLT (Fig. 1).

A further 25 patients underwent PVE followed by re-evaluation of the FLRV before extended liver resection. Out of those 25 patients, extended right hepatectomy was performed in 12 patients after efficient PVE treatment and were categorized as PVEres-group (Fig. 1). PVE treatment was insufficient in 13 patients. Eight of these patients were scheduled for a salvage ISLT procedure to achieve an adequate FLRV, and were analyzed in this retrospective study as ISLT-group (Fig. 1). Five out of the 13 failed PVE patients were not eligible for further surgery due to insufficient FLRV gain or decisive tumor progression and were included as a separate group for analyses (PVEnores-group).

We compared survival time following the date that irresectability was determined after PVE in the PVEnores-group with survival after the last scheduled hepatic surgical intervention in the other two patient cohorts in this study. Further patient characteristics and surgical outcome were compared between the groups.

All patients with locally advanced liver malignancies were discussed and reviewed by a multidisciplinary tumor board including gastroenterologists, surgeons, radiation oncologists, pathologists and radiologists. This study was approved by the local institutional review board (Heinrich Heine University, Duesseldorf, Germany; study-no.: 2018–258-KFogU). All procedures performed in this study were in accordance with the ethical standards in the 1964 Declaration of Helsinki and its later amendments. Informed consent was waived because no data regarding the cases were disclosed.

The following parameters were obtained: patients characteristics including gender, age, ASA (American Society of Anesthesiologists) score, BMI (body mass index) and diabetes, oncological characteristics including tumor type, TNM stage, grading, R-status and neoadjuvant chemotherapy as well as surgical characteristics including hospital stay, morbidity and postoperative 90-day mortality rates, survival, postoperative liver function and radiological data with volume gain of FLRV. Postoperative complications were classified I-IV according to the Clavien-Dindo classification [27].

### Hepatic volumetric assessments and portal vein embolization

Patients underwent routine staging by using preoperative helical computed tomography (CT) scanning of the chest, abdomen, and pelvis. Initial total liver volumes (TLV) that excluded tumor volume (TV) and the FLRV were estimated at time of indication for hepatic resection by CT. Measurements were performed manually with respect to the hepatic segmentation based on the distribution of the portal pedicles and the location of the hepatic veins (Couinaud terminology) as previously described [21, 28]. From week 2 post PVE, differential absolute, relative and daily gains of the FLRV post-PVE were calculated in 2–3 weeks intervals to measure the change in hepatic volume: before PVE, after PVE and before resection. Patients after ISLT received CT volumetry every week.

Two methods for FLRV evaluation extrapolated from liver transplantation literature were applied to evaluate the sufficiency of the FLRV prior hepatic resection. Those are a) the ratio of FLRV to total liver volume and b) the ratio of FLRV to body weight (FLRVbw). In our study, we utilized method b) for determing the threshold of liver volume for resectability to exclude a phenomenon possible in method a) which is comparing parts of normal liver parenchyma to others compromised by biliary or vascular obstruction, by portal vein embolization or disturbance in measurement due to tumor volume as also discussed elsewere [29]. Extrapolating from living-donor liver transplantation, we hypothesized that FLRVbw more accurately assesses the functional limit of hepatectomy. For the FLRVbw, a ratio of of 0.4 to 0.5 was determined to be sufficient [29, 30]. For extensive hepatic resection surgery as performed in this study, we set a rather conservative threshold of 0.5 for volumetric resectability in non-cirrotic patients as proposed also by others [30].

For PVE, a transileocolic portal venous approach was used. After visualization of the portal venous tree, histoacryl or polyvinyl alcohol particles were applied to occlude portovenous branches to liver segments I/IV-VIII as previously described [21]. After the hypertrophy period, an extended right hepatectomy was performed.

### Surgical technique

The surgical technique for ISLT was performed as previously described [21]. In brief, for the ISLT procedure in stage 1, the right portal vein was divided, whereas the right hepatic artery and right bile duct were identified and marked with a vessel loop for later transection during stage 2. After complete liver mobilization and transection of all retrohepatic veins, the liver parenchyma between segments II/III and I/IV-VIII was transected by using a cavitron ultrasonic surgical aspirator (CUSA®, Valleylab, Boulder,Colorado/USA) [21]. In stage 2, the right hepatic artery and right bile duct were transected and the resected liver was removed.

### Posthepatectomy liver failure

Posthepatectomy liver failure (PHLV) was defined as the impaired ability of the liver to maintain its synthetic, excretory, and detoxifying functions, which was characterized by an increased international normalized ratio (INR) and concomitant hyperbilirubinemia according to the normal range of cut-off labels of our local laboratory on or after postoperative day (POD) 5 [31]. When INR or bilirubin were already increased preoperatively, PHLF was defined by increased values on or after POD 5 compared to the values of the previous day as previously described [31].

### Statistics

All data were retrospectively collected and transferred into a database. Statistical analysis and graphing were performed using MS Excel and JMP 14.1 from SAS Institute Inc., Cary, USA. All results are expressed as mean ± standard deviation. Statistical significance was determined by Student’s t test and Chi-Square test. Survival curves significance was defined as *p < 0.05. **p < 0.01. The Kaplan-Meier method was used to estimate survival curves.

## Results

### Patient characteristics

Median age within PVEres and ISLT groups overall was 67 years and was comparable among these two cohorts (PVEres range: 44–81; ISLT range: 49–81 years, *p = 0.9*2) (Table 1). In the ISLT group was 62.5% male [5] and 37.5% female [3] patients. The PVEres group included seven male (58.3%) and five (41.7%) female patients (*p = 0.85*). ASA score (*p = 0.12*), BMI (*p = 0.73*) and diabetes (*p = 0.49*) were equally distributed between the study groups.

**Table 1:** Patient and surgical characteristics comparing ISLT (*n* = 8) and PVEres (*n* = 12) group

	***PVEres***	***ISLT***	***p-value***
	***n*** = 12	***n*** = 8	
**Patient characteristics**			
**Age** years (mean + SD)	67.5 + 11.2	67 + 9.8	*0.92*
**Gender** n (%)			*0.85*
Male	7 (58.3)	5 (62.5)	
Female	5 (41.7)	3 (37.5)	
**ASA score** n (%)			*0.12*
2	6 (75)	2 (33.3)	
3	2 (25)	4 (66.7)	
**BMI** (mean + SD)	26.5 + 3.7	27.1 + 4.2	*0.73*
**Diabetes** n (%)			*0.49*
Yes	3 (25)	1 (12.5)	
No	9 (75)	7 (87.5)	
**Surgical characteristics and early outcome**			
**Hospital stay (d)** (median + SD)	36.3 + 28.2	30.0 + 28.9	*0.63*
**Morbidity (Dindo/Clavien)** n (%)			*0.49*
no complication	4 (33.3)	1 (12.5)	
I-IIIa	2 (16.7)	1 12.5)	
IIIb-IVb	6 (50)	6 (75)	
**Postop 90-day mortality** n (%)	3 (25)	2 (25)	*1*

*ASA* American Society of Anesthesiologists, *BMI* body mass index, *CCC* cholangiocellular carcinoma, *CRLM* colorectal liver metastasis, *d* days, *HCC* hepatocellular carcinoma, *ISLT* patients with in situ split transection along segments II and III plus portal ligation to hepatic segments IV to VIII, *RCLM* renal cell carcinoma liver metastasis, *NET* neuroendocrine tumor, *N* node, *M* metastases, *PVEres* extended right hepatectomy promptly following sufficient PVE, *SD* standard deviation, *T* tumor

### Surgical characteristics and outcome

There was no significant difference between the PVEres and the ISLT groups regarding mean hospital stay (36.3 days +/− 28.2 vs. 30 days +/− 28.9; *p = 0.63*; Table 1). Complication were classified according to the Dindo-Clavien classification [27]. There were no significant differences in minor or major postoperative complications between the ISLT and the PVEres group (*p = 0.49*). Four patients (33%) in the PVEres group and one patient (13%) in the ISLT group had an uneventful postoperative course without any minor or major complication. Minor complications (grade I-IIIa) related to resection occurred in two patients (17%) and one patient (13%) respectively. This included two wound infections and two patients with cholangitis. Major complications (grade IIIb-IVb) occured in six patients in each group (50% versus 75% respectively) (Table 1). Three PVEres-patients (25%) and two patients after ISLT (25%) had postoperative bile leaks. Posthepatectomy liver failure with increased INR or hyperbilirubinemia on or after postoperative day 5 occurred in three PVEres patients (25%) and one ISLT patient (13%) respectively. Two patients (17%) in the PVEres group and one patient (13%) in the ISLT group developed temporary renal failure. One failed hepaticojejunostomy, which needed revision surgery, and one acute respiratory distress syndrome occurred in the ISLT group. In the PVEres group, one small-bowel perforation with intestinal fistula and one case of postoperative bleeding occurred.

The 90-day mortality rate following extended right resection (stage 2 in the ISLT-group) was 14.3% in the ISLT-group (*n* = 1/7; data not shown) and 25% in the PVEres-group (3/12). Overall 90-day mortality subsequent to the last scheduled resection-concept surgery (stage 1 or 2) was 25% in both groups (PVEres: 3/12; ISLT: 2/8; *p = 1*; Table 2). For the ISLT group, one patient died during the split procedure due to a fatal coronary infarction and one patient developed a myocardial infarction on day 7 following resection-completion surgery with lethal outcome. In the PVEres group, two patients died from septic multi organ failure. One patient died as a result of postoperative liver failure. However, there were no significant differences among groups regarding major complications (*p = 0.49*) or regarding the 90-day mortality (Table 1).

**Table 2:** Oncological characteristics comparing ISLT (*n* = 8) and PVEres (*n* = 12) group

	***PVEres***	***ISLT***	***p-value***
	***n*** = 12	***n*** = 8	
**Oncological characteristics**			
**Tumor Type** n (%)			*0.7*
CRLM	3 (25)	2 (25)	
RCCLM	0	1 (12.5)	
HCC	3 (25)	2 (25)	
CCC	5 (41.7)	3 (37.5)	
NET	1 (8.3)	0	
**TNM stage** n (%)			
**T-stage**			*0.88*
1	2 (16.7)	2 (28.6)	
2	2 (16.7)	1 (14.3)	
3	7 (58.3)	3 (42.9)	
4	1 (8.3)	1 (14.3)	
**N-stage** n (%)			*0.31*
0	7 (58.3)	2 (25)	
1	4 (33.3)	3 (37.5)	
2	0	1 (12.5)	
Nx	1 (8.3)	2 (25)	
**M-stage** n (%)			*0.21*
0	7 (58.3)	2 (25)	
1	5 (41.7)	5 (62.5)	
Mx	0	1 (12.5)	
**Grading** n (%)			*0.2*
G2	8 (66.7)	8 (100)	
G3	3 (25)	0	
Gx	1 (8.3)	0	
**Neoadjuvant chemotherapy** n (%)			*1*
Yes	3 (25)	2 (25)	
No	9 (75)	6 (75)	
**R-status** n (%)			*0.34*
R0	11 (91.7)	7 (87.5)	
R1	1 (8.3)	0	
Rx	0	1 (12.5)	
**Number of lesions** (mean + SD)	2 + 1.9	4 + 4.6	*0.36*
**Maximum tumor diameter** (mean + SD)	80.5 + 47.1	66.3 + 27.7	*0.51*

*d* days, *ISLT* patients with in situ split transection along segments II and III plus portal disruption to hepatic segments IV to VIII, *PVEres* extended right hepatectomy promptly following sufficient PVE, *SD* standard deviation

### Oncological characteristics

Diagnosis for extended right hepatectomy showed various tumor types with no statistical difference in our two groups (Table 2). Likewise, neither TNM classification nor tumor grading caused a significant difference between the study groups. 25% of patients (PVEres: 3; ISLT: 2) received neoadjuvant radiochemotherapy (*p = 1*). Local R0 (margin-free) resection could be achieved in 92% (PVEres) and 88% (ISLT) of the resected patients (*p = 0.34*). The mean number of liver lesions was 2.4 for the PVEres group compared to 4 in the ISLT group with no statistical difference. The maximum tumor diameter ranged 31–115 mm in the ISLT group compared to 14–180 mm in the PVEres group with no statistical difference (Table 2).

### Growth of FLRV following PVE +/− ISLT

**Fig. 2:**
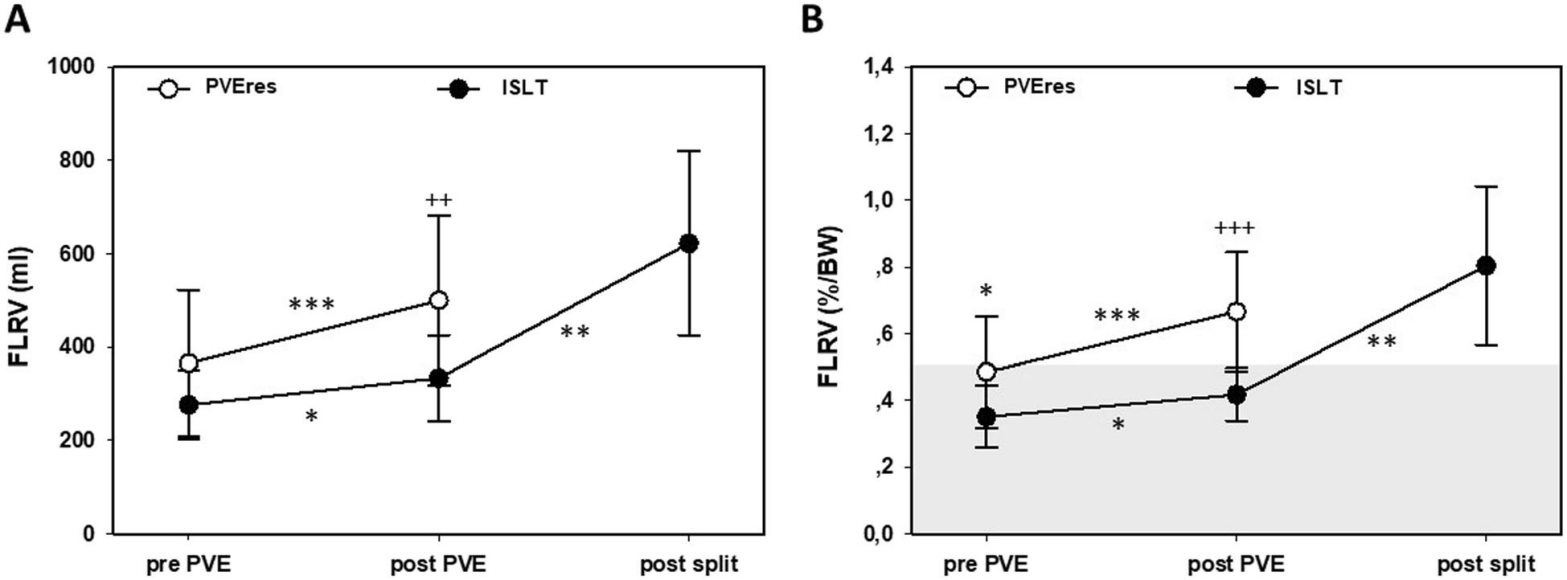
**a**. ISLT (PVE + in situ split + resection) patients demonstrated a significant increase in FLRV post split. **b**. Increase in FLRV to bodyweight ratio post split for ISLT group. The dotted line indicates the critical FLR to bodyweight ratio of 0.5. BW: body weight. FLRV: future liver remnant volume. ml: milliliter. *p < 0.05; ***p < 0.01

The mean initial FLRV prior to PVE was 276 ml + 69 in the PVEres group versus 346 ml + 86 in the ISLT group (Fig. 2a). After PVE treatment, the PVEres patients demonstrated a significant mean increase of 428 ml + 140 compared to ISLT patients with 333 ml + 86. However, seven of the patients who showed an insufficient hypertrophy of the FLRV and who then received a rescue split procedure revealed a significant 2-fold mean increase in liver growth (from 333 ml + 86 to 621 ml + 388) before second-stage surgery (Fig. 2a). This effect was resembled in the % to body weight ratio for FLRV (Fig. 2b). The mean FLRV to body weight ratio (FLRVbw) in the ISLT group was smaller before PVE (0.35 +/− 0.09%) and post PVE (0.42 +/− 0.08%) compared to the PVEres-group (0.49 +/− 0.17% and 0.67 +/− 0.05%) (Fig. 2b). For extensive hepatic resection surgery as performed in this study, we adopted a rather conservative threshold of 0.5 for volumetric resectability in non-cirrotic patients as previously described [30]. Post PVE or post split, we accepted a FLRVbw of 0.4 if an increase in FLRV of at least 20% as a sign of adequate regenerative response was achieved. In seven patients, ISLT was successfully completed with a mean FLRVbw of 0.81 +/− 0.09% receiving a completion of the extended right hepatectomy.

PVEnores patients (*n* = 5) demonstrated a slower growth of the FLRV compared to PVEres patients (*n* = 12) (Suppl. Fig. 1A and B). Four of these patients (pre-ISLT-era of our hepatic resection program) demonstrated inadequate liver growth with an insufficient FLRV. One patient, however, showed sufficient FLRV hypertrophy, but experienced significant tumor progress with peritoneal carcinomatosis as detected during surgical exploration.

### Survival analysis and liver function

**Fig. 3:**
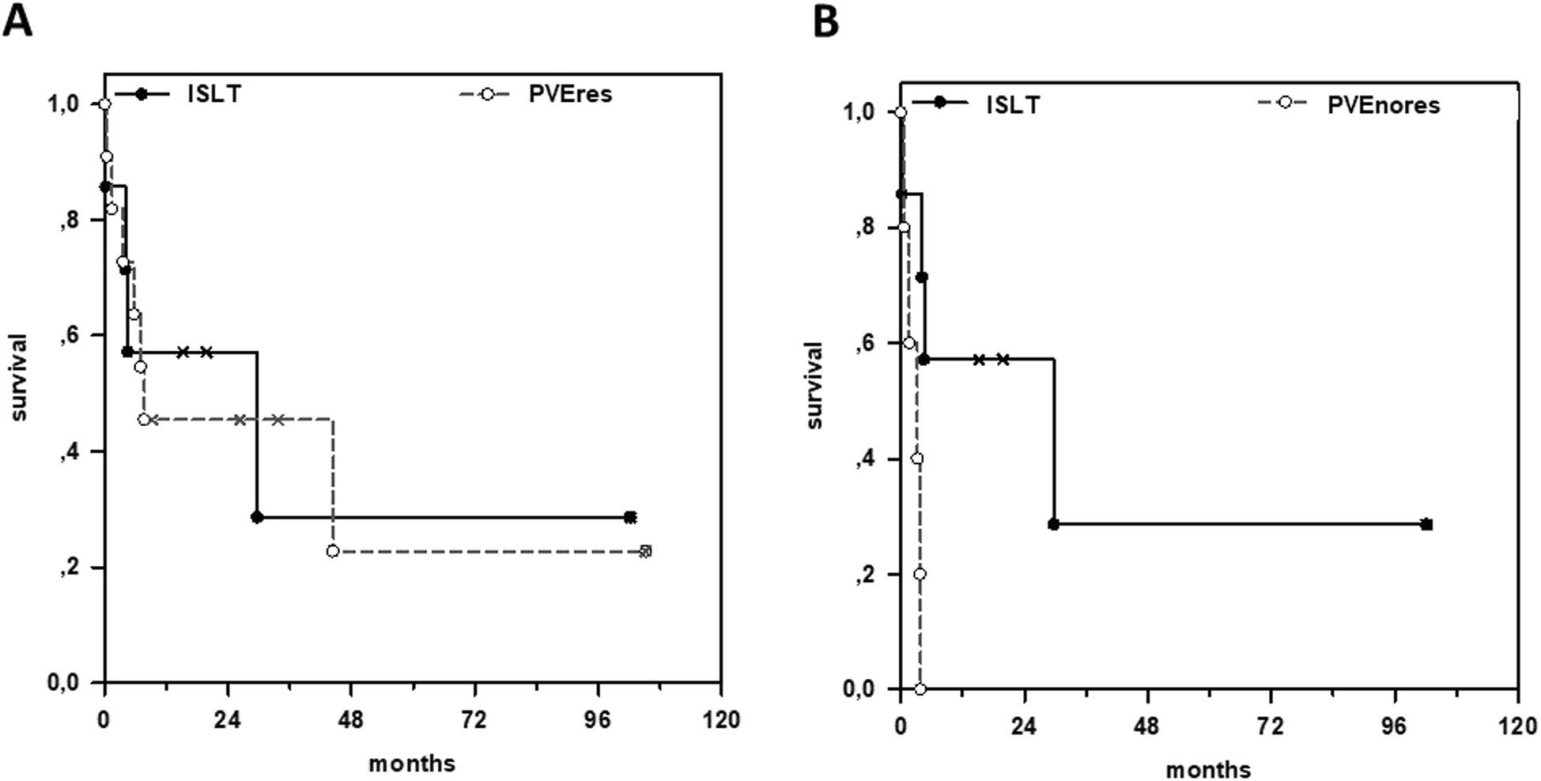
Survival analysis: **a**. Kaplan Meier Survial curve demonstrates similar survival in ISLT (PVE + in situ split + resection) and PVEres (PVE + resection) groups. **b**. ISLT patients show significant longer survival compared to PVEnores (PVE only without resection) patients as demonstrated by Kaplan Meier analysis

With a median follow up of 84.2 months (9.2–132.5 months) overall survival subsequent to the last scheduled hepatic resection-concept surgery was comparable between the ISLT (*n* = 8) and the PVEres group (n = 12) with a 2-year survival of 50 and 45.5% respectively (*p = 0.8*) (Fig. 3a). This is despite conservative data presentation and interpretation with inclusion of the one stage 1 intraoperative mortality in the ISLT group latter due to fatal cardiac infarction. Survival analyses revealed a strong survival benefit for the ISLT group when compared to the PVEnores group. PVEnores patients demonstrated a significantly shorter survival with no patient alive beyond 4 months (*p = 0.03*) (Fig. 3b).

**Fig. 4:**
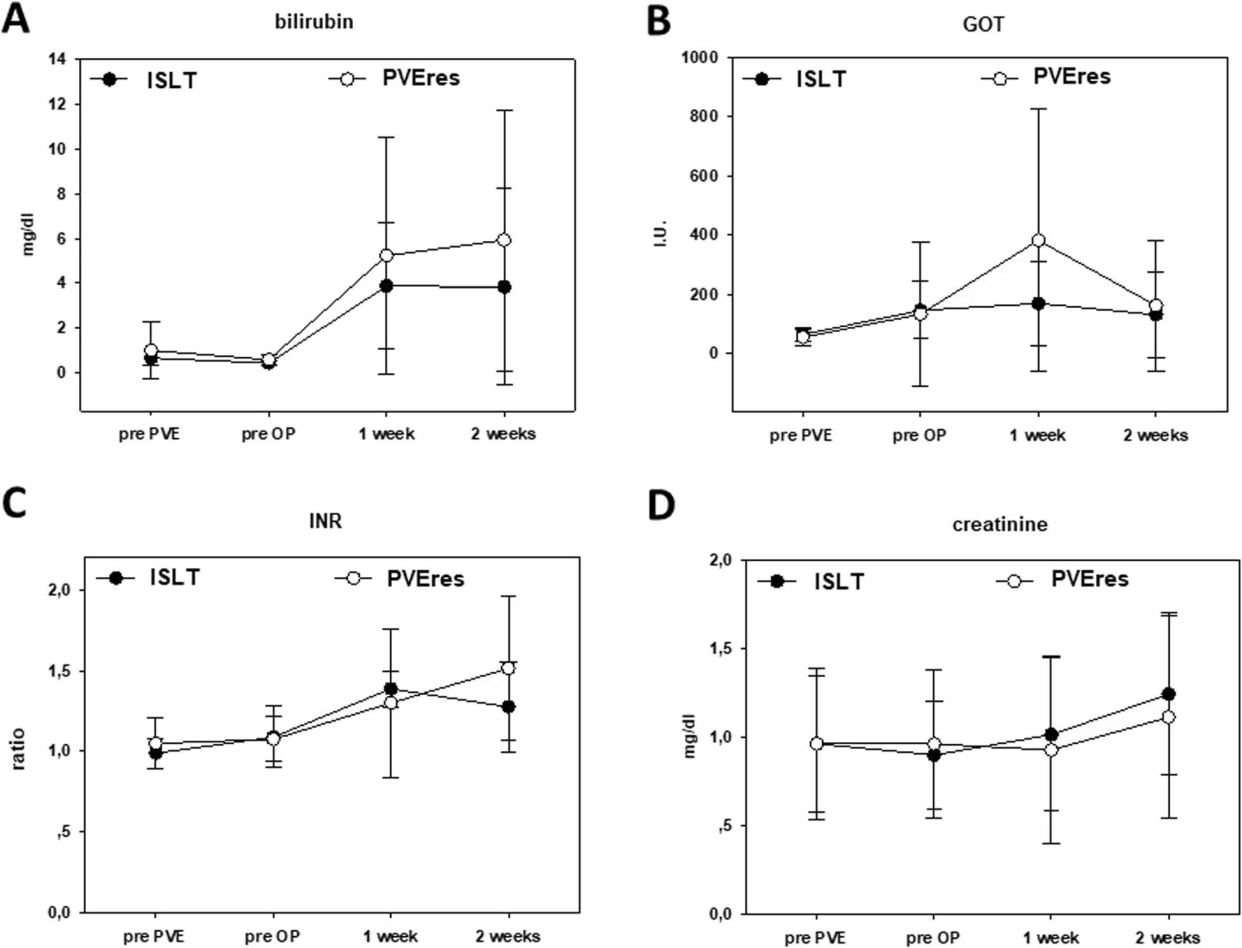
Mean (s.d.) **a**. bilirubin, **b**. glutamate-oxalacetate-transaminase (GOT) and glutamate-pyruvate-transaminase (GPT), **c**. international normalized ratio (INR) and **d**. creatinine levels pre PVE, pre operation, 1 week and 2 weeks after resection for ISLT (PVE + in situ split + resection) and PVEres (PVE + resection) patients. I.U.: international units

Looking at liver function in the clinical course prior PVE, post PVE, and one and two weeks post liver resection, our data did not reveal any statistical differences after ISLT and completion extended right hepatectomy (Fig. 4). Both groups demonstrated a non-significant 5-fold increase in bilirubin as a sign for transient liver insufficiency (prior PVE: PVEres 1 mg/dl + 1.3, ISLT 0.5 mg/dl + 0.3; 2 weeks post op: PVEres 5.8 mg/dl + 5.7, ISLT 4 mg/dl + 4.8) (Fig. 4a). Aspartate aminotransferase levels were slightly increased one week post resection in the PVEres group, but returned almost to normal within two weeks for both groups (Fig. 4b). Both INR and creatinine comparably increased after liver resection one week and two weeks post-op for the two groups (Fig. 4c & d).

## Discussion

This study represents the largest single-center experience with ISLT performed as salvage procedure subsequent to inefficient one-step PVE of all hepatic segments (I/IV-VIII) except II and III. We have previously presented the novel in situ split liver procedure in three patients subsequent to inefficient PVE [21]. In this follow-up study, we demonstrated a comparable outcome after extended right liver resection subsequent to PVE plus ISLT and immediately subsequent to sufficient PVE respectively.

Commensurability of the two groups was demonstrated by comparable patient characteristics including age distribution, co-morbidity and oncological profiles. Our patient population was more heterogeneous from oncologic indications such as CCC, HCC, NET and CRLM; this is in contrast to a recent single center experience on 5 patients solely with colorectal liver metastases (CRLM), who were subjected to rescue ISLT following PVE [4]. This study deviated from our investigation in that only segments V to VIII were embolized. These patients underwent atypical CRLM liver resections in segments I-III in the course of the ISLT surgery (stage 1) and segments IV to VIII in stage 2 surgery, leaving segment I in place. This was also performed in all but one of the 17 patients in the largest series of rescue ISLT subsequent to failed PVE to date reported on a collective derived from an ISLT (ALPPS) database based on 12 hospitals [5]. In this study, PVE was mainly limited to the right portal branch; segment IV was only addressed in one case. Another recent study performed by Ulmer et al demonstrates the largest single-center experience of 9 patients with rescue ALPPS following insufficient hypertrophy after PVE [26]. However, PVE on segment I was not performed in this study either. In our study, we present eight patients with intention to treat subsequent to failed PVE of segments I and IV to VIII. Out of this group, first stage ISLT was followed by second stage extended right hepatectomy (segments I + IV to VIII) in seven cases. Because we have routinely occluded all portal branches to the to-be-resected segments including segment I, which is unique to all previous mentioned studies, we have applied the full potential of PVE with limited residual perfusion of the right side. However, how much influence these portal-venous shunts have on liver growth, needs to be further investigated.

The ISLT procedure is a relatively novel technique, which has evoked a hot debate in the last few years because it is known to be paralleled by high morbidity and mortality rates, especially in patients with primary liver tumors or cirrhosis patients [6, 22, 32–34]. Previous reports suggest that careful patient selection for ISLT is necessary to reduce the mortality [6, 35, 36]. The advantage of ISLT strategies is clearly seen in the high proliferative stimulus that induces hypertrophy of the FLRV at a rate that is unmatched by PVE [17, 37–39]. An inadequate increase in the FLRV after PVE is a challenging and limiting problem which represents an exclusion criteria for extended hepatic resection [2, 3, 34, 40, 41].

The development of left to right portal venous shunting in spite of PVE occlusion has been shown to negatively correlate with FLRV hypertrophy [42, 43]. The improved gain in FLRV subsequent to the ISLT procedures may be a result of the improved isolation of the non-future liver remnant from portal blood supply by transecting the plain for collateral formation along the border of segment IV and segments II /III. Further, sparing segment IV in the course of PVE concepts seems to be associated with compromised levels of FLRV gain [44], which is supported by our observation that PVE with inclusion of segment IV is more effective for increasing the FLRV than open right portal ligation [10]. Although segment IV perfusion via segmental portal branches is not influenced by the ISLT procedure itself, a two-sided isolation of hepatic segment IV - that is the PVE-occluded portal supply from central and collaterals along falciform ligament on the other side - might maximize the FLRV gain after the ISLT. Moreover, it seems advisable to address segment IV during PVE in order to achieve the maximum PVE-effect and to reduce the chance of a failed PVE.

The mean 90-day mortality after extended right resection (stage 2) following ISLT (stage 1) of 14.3% (*n* = 1/7) is in the scope of that reported by others of up to 16% [35]. Lower rates such as 8.8% have been reported in series with patients exclusively treated for CRLM [4]. The ALPPS registry did not reveal any morbidity or mortality rates after rescue ISLT [5]. However, our patient collective demonstrated an average age of 67 years; this might be relevant for the interpretation of the herein reported mortality because patient age beyond 60 years is associated with an increased risk for morbidity and mortality due to impaired regenerative potential [45].

For cases of ISLT as a rescue measure, we and others [4, 5, 23, 26] have demonstrated the preserved capacity to gain the FLRV even with previously failed portal occlusion concepts. Even on a small number of patients, we could demonstrate for the first time long-term outcome on patients after ISLT in this single center analysis. We revealed a comparable overall survival after resection in the ISLT group compared with patients resected subsequent to sufficient PVE. Without the option of ISLT, those patients would have been categorized as inoperable. Our survival data demonstrate a survival benefit of ISLT individuals over patients in our program who did not reach eligibility for resective surgery due to inadequate FLRV. All non-resected patients died within 4 months.

The amount of liver growth does not necessarily reflect the liver function, especially in patients with chronic liver diseases. However, markers of hepatic damage, metabolism and syntheses were comparable in the course following extended right resection in insufficient PVE plus ISLT patients and adequate PVE alone individuals respectively. These data suggest that liver function follows volume subsequent to ISLT as a salvage strategy to pre-condition the FLRV after failed PVE.

Obviously, this study has some limitations. Even though we present a large single-center experience of eight patients in this selected patient group, it remains a small heterogenous collective with patients with different tumor entities especially in the ISLT and non-resection groups. However, it was conducted with standardized and uniform PVE and surgical strategies, which was not the case in other multicenter studies that have reported on that topic so far [4, 5, 23]. Furthermore, due to the heterogeneity concerning oncological background leading to resective surgery it is very difficult to compare patients, although the resection groups here were comparable among each other to that respect. To overcome some of this limitations, multicentered prospective trials with harmonized interventional and surgical concepts are required to reach an acceptable level of comparability and to draw the right conclusions for clinical practice. Facing a rather infrequent phenomenon of a therapeutic concept, implemented in a limited number of liver resection programs, such a study will be challenging, but vital to accomplish. This kind of study may provide criteria and the decision guidance for the optimal timespan between PVE and making the choice whether to proceed with ISLT or to perform a single-stage extended hepatic resection.

## Conclusion

In summary, our results showed similar liver function and comparable overall survival for patients who underwent a rescue ISLT after inadequate PVE compared to those with immediate extended right hepatectomy subsequent to initially sufficient PVE. We believe that ISLT could be applied earlier after failed PVE because the complication rate is not unfavorable and we could demonstrate a clear survival benefit. Consequently, patients should be re-evaluated 6–8 weeks after PVE, and in cases of inadequate FLRV growth, ISLT can be safely considered as an intermediate step to achieve operability.

## Supplementary information

**Additional file 1: Figure 1.** Future liver remnant volume (FLRV) gain pre or post PVE. A. PVEres (PVE + resection) patients significantly increased FLRV when compared to PVEnores (PVE only without resection). **B.** Statistical difference in FLRV to bodyweight ratio within PVEres and PVEnores patients. BW: body weight. FLRV: future liver remnant volume. ml: milliliter. p < 0.05; ***p < 0.01; n.s. - not significant.

## Data Availability

The datasets used and/or analyzed during the current study are available from the corresponding author on reasonable request.

## Abbreviations

ALPPS: Associating Liver Partition and Portal Vein Ligation for Staged Hepatectomy
ASA: American Society of Anesthesiologists
BMI: Body mass index
CCC: Cholangiocellular carcinoma
CRLM: Colorectal liver metastases
CT: Computed tomography
FLRVbw: FLRV to body weight-ratio
FLRV: Future liver remnant volume
HCC: Hepatocellular carcinoma
INR: International normalized ratio
ISLT: In situ split plus portal vein ligation
PHLF: Posthepatectomy liver failure
POD: Postoperative day
PVE: Portal venous embolization
RCLM: Liver metastases from renal cell carcinoma
TLV: Total liver volumes
TV: Tumor-volume
