# Supplementary figures and images for: In situ split plus portal vein ligation (ISLT) – a salvage procedure following inefficient portal vein embolization to gain adequate future liver remnant volume prior to extended liver resection
Source: BMC Surg. 2020 Apr 6;20:63. doi: 10.1186/s12893-020-00721-y (PMC7333278; doi:10.1186/s12893-020-00721-y)

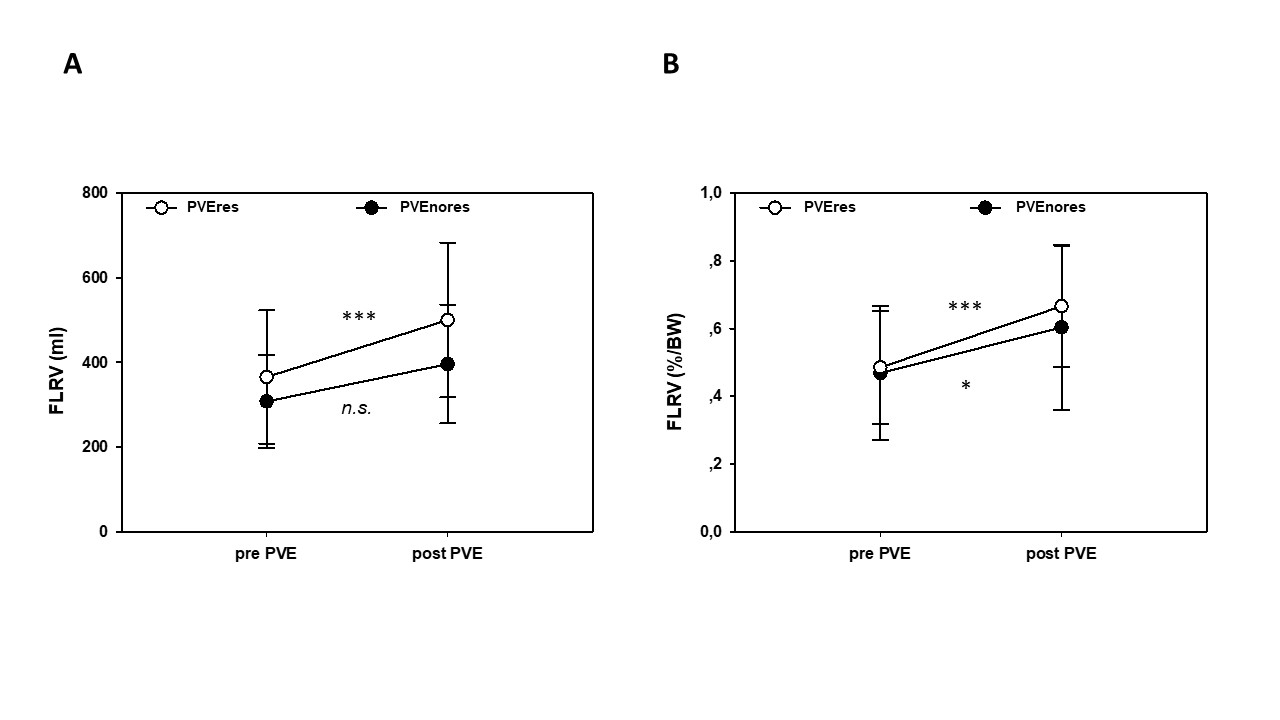

Supplement: Supplementary file 1 — Additional file 1: Figure 1. Future liver remnant volume (FLRV) gain pre or post PVE. A. PVEres (PVE + resection) patients significantly increased FLRV when compared to PVEnores (PVE only without resection). B. Statistical difference in FLRV to bodyweight ratio within PVEres and PVEnores patients. BW: body weight. FLRV: future liver remnant volume. ml: milliliter. p < 0.05; ***p < 0.01; n.s. - not significant. [file 12893_2020_721_MOESM1_ESM.jpg]
